# Noise Evaluation of S-Polymer Gears

**DOI:** 10.3390/polym14030438

**Published:** 2022-01-22

**Authors:** Boštjan Trobentar, Matija Hriberšek, Simon Kulovec, Srečko Glodež, Aleš Belšak

**Affiliations:** 1 Faculty of Mechanical Engineering, University of Maribor, Smetanova 17, 2000 Maribor, Slovenia; bostjan.trobentar@um.si (B.T.); ales.belsak@um.si (A.B.); 2 Podkrižnik d.o.o., Lesarska cesta 10, 3331 Nazarje, Slovenia; matija.hribersek@podkriznik.si (M.H.); simon.kulovec@podkriznik.si (S.K.)

**Keywords:** polymer gears, S-gears, sound, noise analysis

## Abstract

In this study, an acoustic behaviour of S-polymer gears made of the material combination POM/PA66 was investigated and compared to the standardised involute gears (E-gears). Basic evaluating characteristics included noise during operation, which is of particular significance when noise reduction is expected. The measured signals were analysed in time and frequency domains and the levels of acoustic activity were compared. The experimental results have shown that the sound pressure level of both E- and S-polymer gears are proportional to the torque. However, the comprehensive noise evaluation has shown some advantages of S-polymer gears if compared to the E-polymer gears. In that respect, S-polymer gears were found more appropriate for noise reduction of gear drive systems in the case of normal loading and typical drive speed. Future studies in the operating behaviour of S-polymer gears could also cover noise evaluation using new methods of sound signal analysis at different temperatures of gears.

## 1. Introduction

Noise generated in gears can be disturbing and unpleasant. Also, it has an impact on the machine operation. Gear noise is generated due to the engagement of teeth of the gear pair, due to the quality of gears and due to cinematic relations caused by the geometry of the teeth profile. Motion errors and local accelerations occur, which bring about vibrations. Friction is the main difference between steel and polymer gears. Temperature also has an impact in the case of polymer materials, changing the tooth stiffness.

Polymer gears are used in many industries and have various applications, e.g., in office appliances, mechatronic devices, household appliances, medical instruments, computer and laboratory equipment, etc. Classical cutting processes can be used to produce these gears, whereas, for large series productions, injection moulding is frequently used. Some of the main advantages of polymer gears include high specific mechanical properties (high size–weight ratio), good tribological properties (low coefficient of friction, self-lubrication), high resistance against impact loading, etc. In addition, they can absorb and dampen vibration and diminish noise. Also, it is possible to use them in wet environments and in areas intended for preparing food, etc. However, polymer gears have some disadvantages as well. For instance, they have a lower load-carrying capacity and can operate at lower temperatures in comparison to metal gears. Also, with polymer gears, it is more difficult to achieve high tolerances (especially in the case of moulded gears, etc.). [1,2,3,4,5]. Recently, reinforced polymer gears have been used increasingly to improve the polymer for better heat resistance and higher strength in gears [6,7,8]. Treviso et al. [9] reviewed damping in composite material with a different model. Goriparthi et al. [10] analysed the performance evaluation of composite gears by varying weight function, and they confirmed a positive influence of composites on mechanical properties. Gnatowski et al. [11] presented an analysis of the impact upon the modification of thermomechanical properties of polymers on the gear meshing teeth process. The experimental results showed positive changes in the material strength for PVDA (Tecaflon) and PE (Polyethylene) after heat treatment. 

Gears are very important parts of many mechanical systems for power and rotational movement transmission. Due to their failure, the entire system can stop operating. Consequently, it is of key importance to assess their load capacity against failures, taking the loading conditions into consideration when the dimensions of gear drives are decided upon. When it comes to polymer gears, the standardised procedure is usually based on the VDI 2736 standard [12]. The load-carrying capacity calculation details of polymer gears are available in the aforementioned standard, with some special characteristics of polymer materials being taken into consideration. 

Polymer gears are often used without lubrication. As a result, a gear drive fatigue life can be relatively short due to high contact friction, which leads to a high degree of wear. This applies primarily to medium- to high-power transmission systems. Singh et al. [13] studied how many polymer materials that are usually used in polymer gearing applications behave in regard to wear. According to their conclusions, the maximum specific wear rate applies for ABS (Acrylonitrile Butadiene Styrene) gears, whereas the minimum specific wear rate is achieved with POM (Polyoxymethylene) gears. In another work of Singh et al. [14], the wide influence on polymer gear behaviours under different conditions was analysed. Design and modification features of polymer gears for the improvement of the performance and durability were also described. Li et al. [15] focused on the engagement behaviour of teeth flanks of polymer gears. According to the results of their experiments, wear can be reduced significantly by modifying the tip relief of gear flanks. Lin and Kuang [16] studied the dynamic interaction between contact loads and tooth wear during the engagement of polymer gears. With their numerical analyses, they have indicated that tooth wear can observably change the dynamic load histogram of an engaged polymer gear pair. Mao et al. [17] addressed the impact that the selection of the manufacturing process (machine cutting or injection moulding) has upon the wear and contact behaviour. Following the discovery that the manufacturing process had no influence on the wear rate, they concluded that machine cut acetal gears can be designed with the existing methods used for injection moulding of acetal gears. The influence of shrinkage on the dimension of small polymer gears was investigated by He et al. [18]. The nonlinear response of shrinkage confirmed the study, and the authors solved the problem with a cooling rate. Pisula et al. [19] investigated the wear of polymer gears, made with additive technology. Different additive techniques were analysed and tooth surface topography was monitored. They concluded that PEEK (Polyetheretherketone) is resistant to wear related to ABS and PEI (Polyetherimide). Dearn et al. [20] also investigated the wear behaviour of polymer gears. They studied the influence of using various surface coatings on controlling friction and wear of PEEK and PA (Polyamide) polymer gears. According to their results, friction and wear were diminished most significantly when using the PTFE (Polytetrafluoroethylene) coating for both PEEK and PA polymer gears. 

In comparison to metallic gears, the operating temperature of polymer gears is lower. Kalin et al. [21] determined the operating temperatures of gear flanks. They discovered that, due to the operating conditions, the temperature changes considerably. According to their conclusions, it is not possible to neglect the gear temperature control when testing the wear and fatigue of polymer gears. For defining the temperature area of polymer gears while they operate, a new numerical approach was suggested by Roda-Casanova et al. [22] and Raghuraman et al. [23]. Their findings were confirmed with an experiment. Jena et al. [24] proposed a comprehensive review of the tribological performance of polymer gears. The authors concluded that tribological (coefficient of friction) and process parameters (temperature, applied torque, speed) play an important role when deciding the friction and wear behaviour of polymer gears during their operation.

Numerous research studies about the noise of steel gears were conducted. However, the sound characteristics of polymer gears with a new gear profile are relatively new. For noise reduction in a gear system, Liguori et al. [25] used the numerical approach to describe how stresses resulting from the meshing of gears affect the acoustics emission. Different parameters were taken into account, such as friction, material and lubrication. Results indicated that by changing the material from hard to ductile, the gear noise was improved, while by increasing the rotational speed or friction, the acoustic emissions were increased. Sight et al. [26,27] investigated polymer spur gears with various functionally graded materials, influencing tribology properties and noise emission. According to their conclusions, the noise reduction was better with gears produced by injection moulding. Nozowa et al. [28] studied the tribology of polymer gears and steel gears. Based on an experiment, noise reduction was observed and the influence of low adhesive strength against shear was confirmed. The influence of meshing stiffness on the sound emission of a gear pair was studied by Malakova et al. [29]. A significant individual parameter that is a source of vibration and noise was found in the stiffness of the gearing. Sun et al. [30] presented gear noise from a source-path-receiver mechanism and precisely predicted noise from its source. The excitation of gear noise was categorised into a few main groups according to the geometrical orientation of a gear. Sharma et al. [31] investigated noise and damping of polymers and composite spur gears and concluded that polymers and reinforced polymers were a good solution for light-load drive systems with low noise. Vibration fault detection of polymer gears was studied by Kumar et al. [32]. For the identification of failure, the statistical feature of the acquired signal was used. With high order statistics indicators, the failure was successfully detected. Radionov et al. [33] investigated the acoustic characteristics of a gear pump with a polymer pinion shaft. They confirmed that polymer gears and shafts had a better acoustic feature than steel gears. Research in preliminary fatigue tests of different polymer gears with different profiles was made by Sobolat et al. [34]. According to their conclusions, in the range of nominal load, the sound intensity level and temperature of the meshing zone was changed with a different profile. Some material combinations gave useful results. Varun et al. [35] analysed lightweight polymer composite spur gears. Various types of reinforcements and fillers were used in polymer gears in order to enhance performance. Tests were carried out at different torque levels and speeds, and the number of cycles was monitored. Poongodi et al. [36] analysed failure in nylon gears using the sound analysis technique, based on parameters such as kurtosis, RMS, variance and standard deviation value. Faultless and defective gears were compared and significant changes in sound signal were discovered. 

However, the noise characteristics of polymer gears published so far mainly refer to involute gears. Only a few published works (Duhovnik et al. [37], Hlebanja et al. [38] and Trobentar et al. [39]) deal with the failures of polymer S-gears. That is why an extensive experimental investigation on the noise analyses of polymer S-gears is presented here. Section 2.1 briefly presents the theoretical background of S-gears. Section 2.2 describes the testing procedure, while the gear design and material selection are presented in Section 2.3. The theoretical background of the noise evaluation in frequency and time-frequency domain is explained in Section 2.4 and Section 2.5. The experimental results in Section 3 performed with S-gears are compared with the results of tests with standardised involute E-gears. The final findings are summarised in the Conclusions.

## 2. Materials and Methods

### 2.1. Theorethical Background of S-Gears

S-gears were developed to replace involute gears in industries, where serious scuffing in addendum and dedendum is present. Modern gears are developed on the basis of the curved contact path, where forced convex–concave is present in the beginning and at the end of meshing [38]. With convex–concave, the load during the contact is significantly reduced and such gears are operational significantly longer. The rack profile of the cutting tool for S-gears is analytically defined in Equation (1) [40,41].

(1) $$y_{P}=a_{P}\left[1-{\left(1-x_{P}\right)}^{n}\right]$$

The upper part of the rack profile is defined using a parabolic function, though its counterpart is derived as a half-symmetric function. The rack flank shape is defined by height factor *a_P_* and curvature exponent *n*, and module *m* is used as a unit of measurement. *y_P_* and *x_P_* are the rack flank coordinates with the origin in the kinematic pole C. Such a rack profile defines a unique path of contact and out of there, gears with any number of teeth can be derived with a bijective transformation.

**Figure 1:**
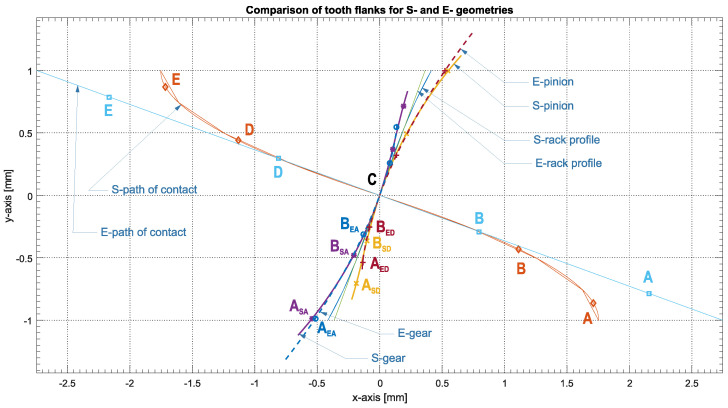
Comparison between the meshing of S- and E-gears.

Characteristic comparisons of S-gears and E-gears (involute) with the same number of teeth *z*_1_ = *z*_2_ = 20 and modules *m* = 1 mm are depicted in Figure 1. The difference in length of addendums for both gears can be recognized when compared with its dedendum. In both examples, the dedendum is shorter than the addendum. Further, when S- and E-dedendums are compared, it is possible to clearly see the differences in length. The S-dedendum is significantly longer than the E-dedendum, (A_ED_C) < (A_SD_C). The dedendum length changes in relation to the number of teeth and with the pressure angle. By lowering the number of teeth, the dedendum is shortened, whereas by increasing the contact pressure angle, it is lengthened; in both cases, the addendums become comparatively longer, as seen in Figure 1. During meshing, both gears, E- and S-, exhibit sliding and rolling. The bulk of it is driven by the difference in the flank length between the contacting dedendum and addendum. This is called the contact point density. The contact starts slowly in the dedendum and speeds up in the addendum part. When compared, it can be noted that E-gears produce more sliding and, consequently, more heat than S-gears [42].

In [42,43], Hlebanja also summarised the most important features of S-gears with regard to the E-gears:

1 The tooth flank shape can be optimised by a height factor *a_P_* and exponent *n*. In this way, one could define a stronger tooth root, a longer convex-concave area, curvature of the path of contact, a lower starting pressure angle in C, etc. to achieve the appropriate gearing properties.
2 Spur S-gears can successfully operate with as low as six or even four teeth. Size optimisation of a gearing can be achieved in this way.
3 S-gears feature convex–concave contact near where the meshing starts and ends. This implies higher reduced radii of curvature and results in a lower contact pressure.
4 S-gears also feature a relatively longer dedendum part of a tooth flank (comparing to E-gears). Thus, in general, one could observe less sliding of a contacting pinion dedendum and gear addendum, which results in less friction and in a lower temperature.

### 2.2. Experimental Test

**Figure 2:**
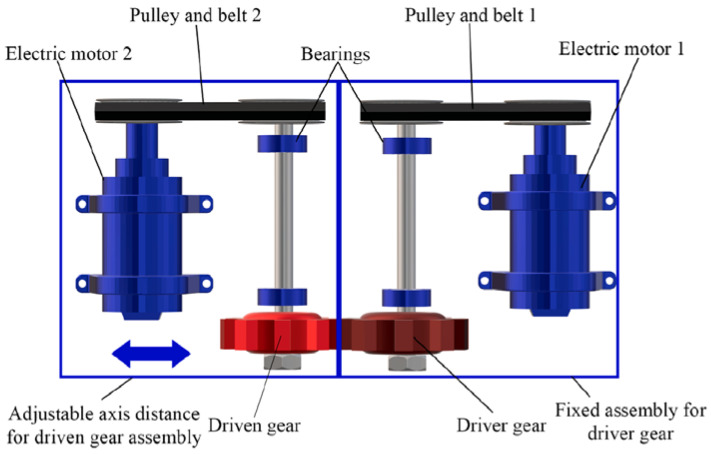
Gear test device (schematic figure).

The testing device was engineered with the aim to test small gears, as seen in Figure 2. The design enables positioning of noise and/or temperature measurement devices. With electric motors that are driven with frequency inverters, the constant torque load can be flexibly adjusted from the driver gear to the driven gear. Power is sent with a toothed belt from the motor to the pulley. The toothed belt enables some damping of vibrations in the system. Torque is achieved by spinning the driven gear more slowly in regard to the driver. One motor is fixed on the frame, while the other is mounted on a high precision adjustment mechanism to allow for accurate setup of axis distance.

On the test bench, primarily the time that elapsed until an event occurred was measured. Three rotational frequencies were selected: 1599 rpm, 1579 rpm and 1547 rpm, which resulted in torque values of 1.1 Nm, 1.3 Nm and 1.5 Nm, respectively. The material combination of POM/PA66 was tested at room temperature (25 °C) and without lubrication. Table 1 shows frequency inverters set up for driven motors. The motor for the driver gear runs at 40Hz (1706 rpm), while the driven motor runs at a lower frequency, thus resulting in the applied torque for the gear pair. 

### 2.3. Gear Design and Material Selection

For gear geometry, the standard involute gears (E-gears) and specially designed S-gears were selected. For E-gears, a standard pressure angle α = 20° was used without any profile shift, therefore *x* = 0. For S-gears, an equivalent gear design was used, with selected parameters shown and compared in Table 2. In testing, thermal expansion of material was neglected, meaning that no centre distance compensation was used, and the value was set as calculated at 20 mm.

Two different polymer materials were used for test gears. Tribological tests [44] prefer material combination POM/PA66, which is also a very common choice among gear design engineers. The first abbreviation always refers to the driver gear (POM) and the second to the driven gear (PA66). Selected materials:

- Polyamide 66 (PA66-HT, TECAMID 66 natural, Ensinger)
- Polyoxymethylene (POM-H, TECAFORM AH natural, Ensinger)

A polymer/polymer gear pair using POM-H as a driving gear and PA66-HT as a driven gear was tested and analysed. The commercial name of the POM-H is Delrin 100 NC010 and it represents a high viscosity acetal homopolymer. The second polymer PA66- HT is known commercially as Zytel 103 HSL NC010 and it represents a polyamide with heat stabilisers for improved temperature resistance. The material properties of both polymers at normal conditions are presented in Table 3.

**Figure 3:**
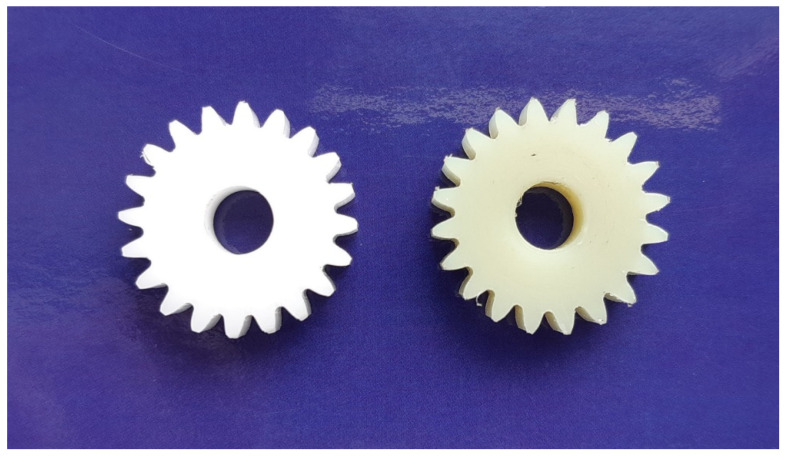
The driver gear (POM) and the driven gear (PA66) of a gear pair.

For preliminary testing, machined gears were used. By using machined gears, greater accuracy of test gears was easily achieved in comparison with injection-moulded gears. All tested gears were fully measured, using an appropriate measuring technique and, thus, the prescribed quality was achieved when producing them, seen in Figure 3.

**Figure 4:**
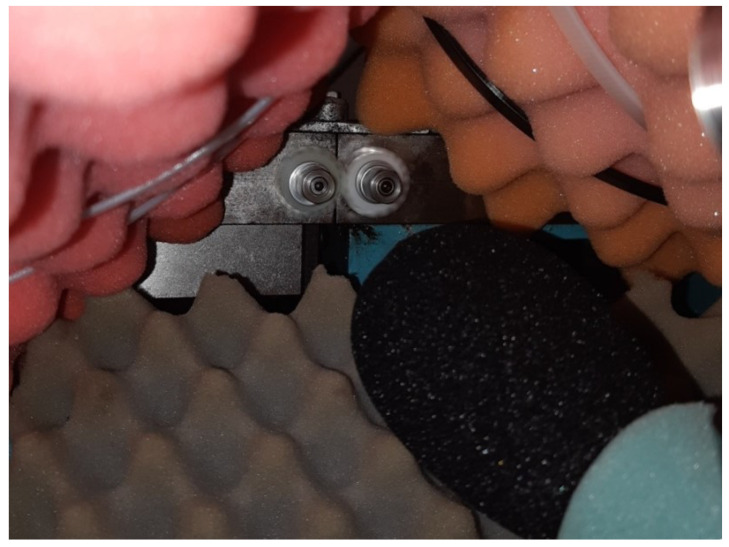
The operated gear pair on the testing device with the soundproof acoustic foam for acoustic soundproof insulation.

To minimise the noise emitted by the electric motor, bearings and toothed belt with pulley, the test gears were protected with soundproof acoustic foam. The tested gears were operated like in a semi-anechoic chamber, which is shown in Figure 4.

For sound pressure measurements, the National Instruments NI PXI 4472 system and the AP 7046 microphone with PS9200 power supply were used. The microphone was placed near the tested gears (100 mm). For signal analysis, the LabView software was used. The acquired signals were analysed in time, frequency and time-frequency space by a developed program, based on a LabView professional version of the software. 

**Figure 5:**
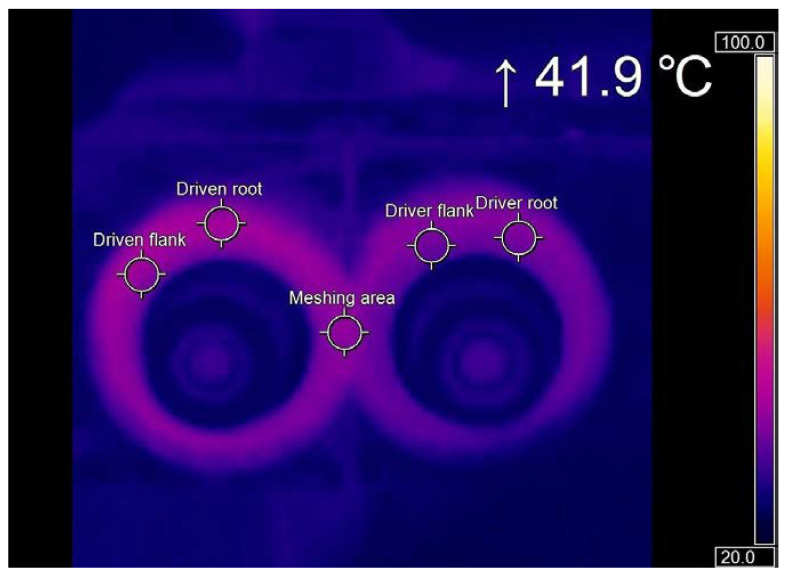
Thermal image of operated gear pair with temperature of meshing area (41.9 °C).

During testing, infrared image camera Optris Xi80 was used to measure average spot temperature in gear pair contacts. The infrared camera had the following specifications: frame rate 50 Hz and display frame rate 20 Hz. The device was connected with a PC, where the thermal state of the gears was displayed and monitored. The temperature of gears during the test was between 40 °C and 43 °C, Figure 5.

All tests were made in the representative number of measuring tests. Also, all gears were checked for their quality before setting them on the testing device. During assembly, they were carefully mounted on the shaft of the testing device and driven (operated) to the appropriate operating temperature. The time required for the running-in phase in the life cycle of a gear pair was about 30 min. With the infrared image camera, we measured the temperature in the meshing area and tooth flanks in real-time. After fulfilling the conditions mentioned above, we performed noise measurements of sound signals.

The Sound Pressure Level is the sound pressure expressed in decibels (dB) so as to compress the large range of Pascals that can be heard in order to make them fitter to be presented.

### 2.4. Freqency Analysis

If *L*^2^ (R) denotes the space of square-integrable functions *x*(*t*), which are measurable, defined in R = (−∞, +∞), it represents analogue signals with finite power. The Fourier transform of a function *x*(*t*) is defined as [44]:

(2) $$F\left[x\left(t\right)\right]=X\left(f\right)=\overset{\infty }{\underset{-\infty }{\int }}x\left(t\right)e^{-j2\pi \mathrm{ft}}dt$$

A Discrete Fourier Transform (DFT) is required for application. The numerical integration of Equation (2) can be used for obtaining it as follows:

(3) $$X\left(f_{k}\right)=\overset{N-1}{\underset{i=0}{\sum }}x\left(t_{i}\right)e^{-j2{\pi f}_{k}t_{i}}\left(t_{i+1}-t_{i}\right)\;k=0,\;1,\;\ldots ,\;N-1$$

Perfect gear sound signals are periodical. As a result of non-linearities in the meshing process, harmonics of the meshing frequency are also included in the signal spectrum. In reality, gears are never perfect. Teeth spacing is usually not a perfect constant and, as a consequence, the contact point oscillates.

### 2.5. Time-Freqency Analysis

In relation to signals of technical analysis, some frequencies occur only in some cases. By classical frequency analysis of such signals, it is not possible to establish the time when particular frequencies appear in the spectrum. Time-Frequency Analysis (TFA) is applied to establish how frequencies of nonstationary signals change with time, and how intense they are [45].

The idea of Short Time Fourier Transformation (STFT) is to divide a time signal into short time intervals first and, after that, to perform frequency analysis of each interval separately. STFT is a linear time-frequency transformation. A method for eliminating defects of Fourier transformation is to compare signals with elementary functions, defined in time–space and in frequency space.

The Fourier transform of the signal *x*(*t*) is not adequate for the frequency domain analysis if it is non-stationary. Local observation of the signal is necessary. In relation to that, the signal has to be divided into segments before carrying out the Fourier analysis. It is assumed that, within each segment, the signal is stationary. Such a signal (divided into segments) is called a windowed signal:

(4) $$x_{w}\left(t\right)=x\left(t\right)\cdot w\left(t\right)$$

The result of the Fourier transform for such a signal can be referred to as a windowed Fourier transform, as it is a function of frequency and windows position:

(5) $$\mathrm{STFT}\left(f,\tau \right)=X_{w}\left(f,\tau \right)=\overset{\infty }{\underset{-\infty }{\int }}x\left(t\right)\cdot w\left(t-\tau \right)e^{-j2\pi ft}dt$$

The selection of the window function *w*(*t*) is possible in such a way that its Fourier transform *W*(*f*) is also a window function. The windowed Fourier transform presented in Equation (5) is often termed as the STFT. The square of the modulus of the STFT is called a spectrogram in engineering applications. For each position of the window, different spectra may be obtained; the total number of these spectra is a function representing a time-frequency distribution.

## 3. Results and Discussion

In general, noise is generated from the meshing process between the teeth of the gear. During the rotational motion of gears, teeth flanks slide and roll and, at the same time, owing to load, i.e., forces, teeth deflection occurs due to stiffness changes within the mesh. Dynamic behaviour of teeth deflection causes vibration, which is a fundamental source of the noise. Additional noise is generated by geometrical errors and different failures of gears. Directly generated gear noise is an aero-acoustical type of noise.

**Figure 6:**
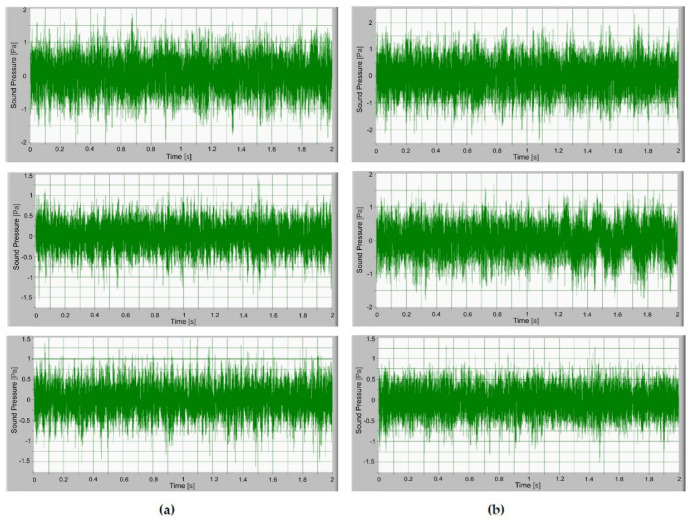
Time signal of sound pressure for gears loaded with torque 1.5 Nm, 1.3 Nm and 1.1 Nm: (**a**) S−polymer gears, (**b**) involute polymer gears (E−gears).

After fulfilling the previously mentioned procedure, the noise of the operating gears was measured as a sound signal. Here, the temperature and speed of the gears were monitored. In Figure 6, time signals of the sound pressure are presented. The amplitude of the sound signal with a lower torque is smaller and increases with the torque. The sound pressure of S-gears is lower than the sound pressure of E-gears. The amplitude of the sound level increases with the torque as can be observed. On average, the temperature during the test was 42 °C.

Before the start of noise measurements, the microphones were calibrated. So, it was possible to determine the appropriate sound pressure level without distortion due to the accuracy of sound measurement. The microphone was used in accordance with the manufacturer’s recommendations regarding its distance and direction of orientation. The operation of electric motors via frequency converters was performed in such a way that the electroacoustic disturbances were the smallest in relation to the design of the test device. The soundproof foam was used to separate the measuring space from the surroundings (see Figure 4).

**Figure 7:**
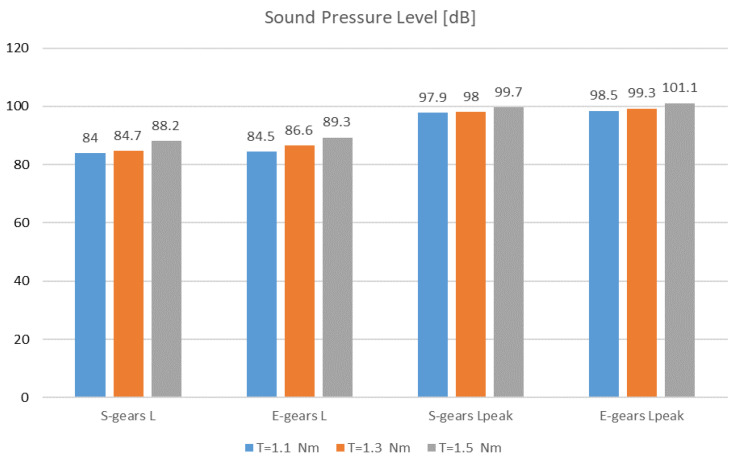
Sound pressure levels L and L_peak_ of E-gears and S-gears for different torques.

In Figure 7, the sound pressure level L and L_peak_ of E-gears and S-gears for different torques are presented. The sound signal was acquired in the phase of normal gear operating in the test stand. The amplitude of the sound pressure level of S-gears is lower than the amplitude of E-gears and it increases with the torque. At the torque of 1.1 Nm, the difference was 0.5 dB, at the torque of 1.3 Nm, the difference was 1.9 dB and at the torque of 1.5 Nm, the difference was 1.1 dB. The aforementioned values apply for the running-in phase and wear-in phase in the life cycle of a gear pair. When comparing the maximum or peak sound pressure level in the phase of the life cycle of increased surface degradation and wear just before the final failure of the gear, at the torque of 1.1 Nm, the difference was 0.6 dB, at the torque of 1.3 Nm, the difference was 1.3 dB, and at the torque of 1.5 Nm, the difference was 1.4 dB. Also, when comparing the peak sound pressure level, S-gears had a lower amplitude. In the running-in phase in the life cycle of a gear pair, the temperature achieved the operational value. Thus, in the running-in phase, i.e., in the beginning, the sound pressure level was slightly larger than when operating in the wear-in phase in the life cycle. By increasing the operating temperature, the noise was slightly reduced in the wear-in phase. Then, the sound pressure level increased significantly even by more than 10 dB in the phase of the life cycle of increased surface degradation and wear just before the final failure of the gear.

In the frequency analysis, it is necessary to ensure a measured signal with a stable rotational speed, so it is necessary to monitor the rotational speed of gear pairs. In the case of deviation of the rotational speed, some problems occur regarding the frequency analysis. Frequency components and their side frequencies that do not reflect the operation of the gear pair may appear. In the test, the maximum deviation of the rotational speed at different torques was 0.45%, which does not significantly impact the frequency analysis.

The measured sound signal was processed with Hamming windows and analysed with frequency transform—FFT, without filters. The sampling rate of acquiring the signal was 65.5 ksample/s.

**Figure 8:**
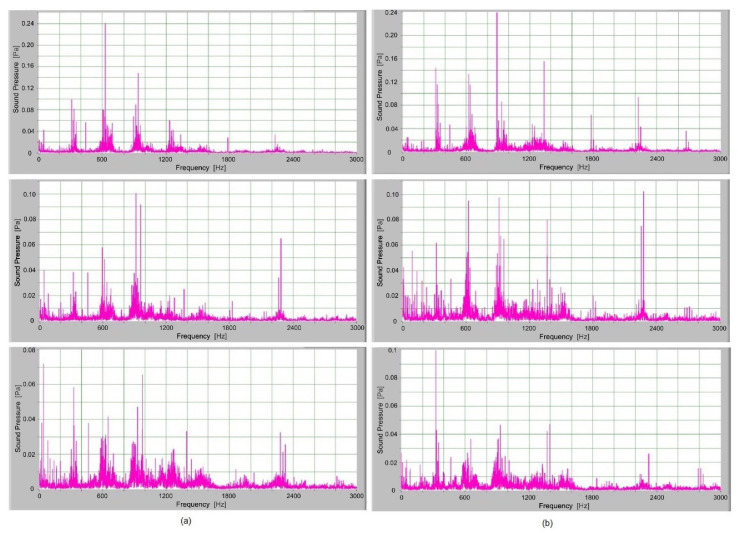
Frequency spectrum of sound pressure for gears loaded with torque 1.5 Nm, 1.3 Nm and 1.1 Nm: (**a**) S-polymer gears, (**b**) involute polymer gears (E-gears).

In Figure 8, the frequency spectrum of the sound signals of S-gear and E-gear with different torques are presented. The meshing frequency and high harmonics are also observed. Also, sidebands around the previously mentioned typical frequencies at 300 Hz, 600 Hz and 900 Hz are noted.

When comparing the frequency spectra, the amplitudes of dominated frequency components of S-gears are lower. The sidebands around the meshing frequency and high harmonics components increase significantly for E-gears. Also, some added frequency components are noted in the frequency spectrum of E-gears with sidebands around the first five harmonics. The peak of dominated frequency components increases with load, and it is higher for S-gears.

The high-frequency components up to the 5th harmonic have a lot of sidebands, particularly E-gears. These sidebands are not a consequence of the meshing frequency, but a semi stochastic phenomenon connected with tooth flanks roughness and friction. Increased roughness and friction produce more side frequency components. Roughness and friction on tooth flanks are semi periodical and a little different for each tooth, which represents the origin of a wider set of side frequency components.

In relation to the time-frequency analysis, the length of the measured signal was 2 s, the frequency sampling was 65.5 ksample/s and the window length was 100 ms. The measured sound signal was processed with Hamming windows and analysed with frequency transform—FFT, without filters. 

**Figure 9:**
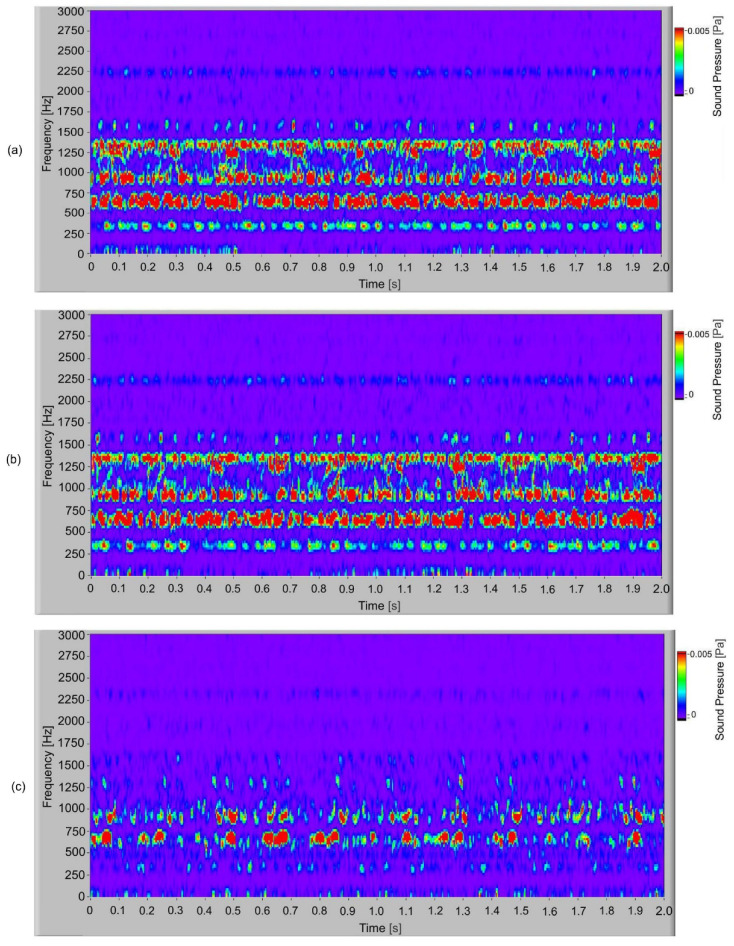
Frequency spectrogram of sound signal for S−gears: (**a**) *T*= 1.5 Nm; (**b**) *T* = 1.3 Nm; (**c**) *T*= 1.1 Nm.

**Figure 10:**
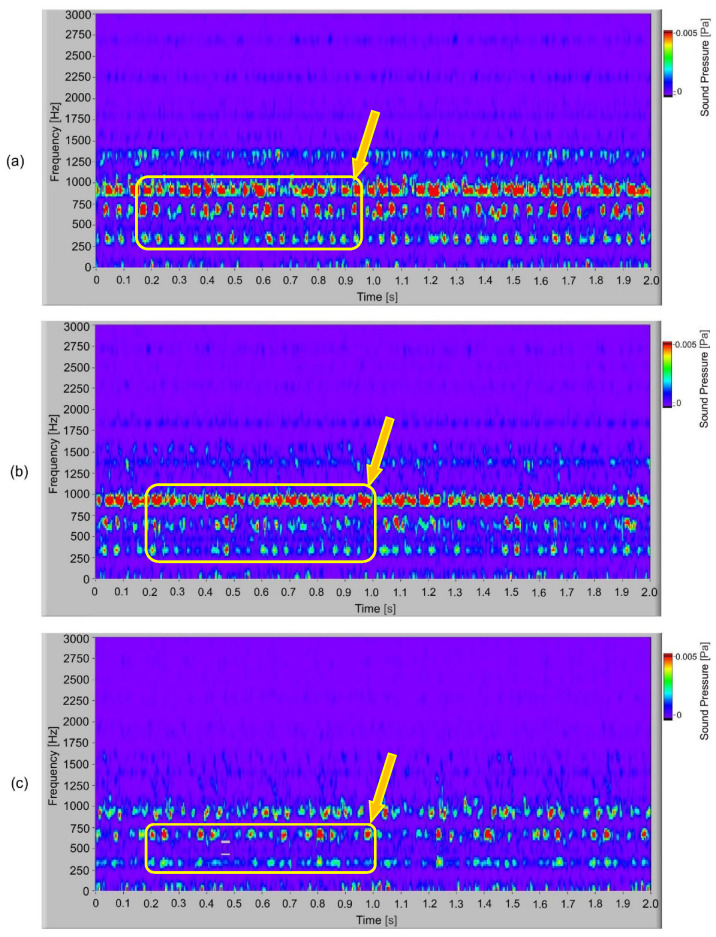
Frequency spectrogram of sound signal for E-gears: (**a**) *T* = 1.5 Nm; (**b**) *T* = 1.3 Nm; (**c**) *T* = 1.1 Nm.

In Figure 9, the frequency spectrogram of the sound signal of the S-gears with different torques is presented. When the frequency spectrum is analysed, the typical frequency components present are observed. In the spectrogram, it is possible to observe how frequency changes in time. In the time–space at low torque, partially stochastic changes of frequency amplitude can be noted. At torque 1.5 Nm and typical frequency components 300 Hz, 600 Hz and 900 Hz with a low amplitude, partial quasi periodical changes during the time can be observed. In Figure 10, the frequency spectrogram of a sound signal of the E-gear with a different torque is presented. At low torque (1.1 Nm), there are quasi periodical changes of dominated frequency components at 300 Hz, 600 Hz and 900 Hz, which, when increasing the torque, change to periodical changes or pulsation of dominated frequency components at 300 Hz, 600 Hz and 900 Hz.

If, in the frequency spectrum, the influence of roughness and friction is observed, the presence of pulsation in time–space in many typical frequency components is observed in the spectrogram. The intensity of pulsation is connected with friction, because the effect of moving and stopping the tooth flank on the surface contact, i.e., the slip-stick effect, is dominant over roughness as the quality of tooth is relatively good and the gear pair operates with normal test parameters.

## 4. Conclusions

This paper presents an extensive experimental study on the noise emission analyses of polymer gears considering a usual material combination POM/PA66. The gears analysed were made of two different tooth profiles. Standard cutting tools were used to machine S-gears and involute gears (E-gears), which were tested afterwards using a special custom-made testing device. The results for S-gears obtained during the experiments were also compared with the results of standardised involute E-gears.

On the basis of a theoretical study and extensive experimental tests, it is possible to draw the following conclusions:

- In many applications, including household and office appliances, gear systems, etc., S-polymer gears could be used. In addition to a stronger tooth root and a longer convex–concave area of meshing teeth, a lower starting pressure angle at point C, assuring suitable gearing characteristics, can also be achieved by optimising the tooth flank shape of S-gears with certain correction factors (height factor *a_P_*, curvature *n_x_*).
- Sound pressure levels of S-gears are lower than those of E-gears. During the meshing, both gears, E-gears and S-gears, exhibit sliding and rolling. S-gears also feature a dedendum part of a tooth flank, which is relatively longer if compared to E-gears. It is possible to observe slighter sliding (slip-stick effect) of a contacting pinion dedendum and a gear addendum, which results in less friction.
- Generally speaking, the sound pressure level of polymer gears is proportional to the torque. In the frequency spectrum, the fundamental frequency and its harmonics increase with load. The influence of friction with micro slip-stick effect on tooth flank causes a stohastic presence of many frequency components in the entire frequency range, particularly between 1–1.6 kH. In the spectrogram, more pulsation can be noticed caused by more sliding in the tooth flank in E-gears. With the load, pulsation of frequency components in the spectrogram increases. Time-frequency spectrograms are useful for the identification of the presence of frequency components in time.
- The results of tests performed with both tooth profiles (S-gears and E-gears) and different torques have revealed that S-gears are more appropriate for the noise reduction of gear drive systems than E-gears in the case of normal loading and typical drive speed.

With reference to the explanation above, it is possible to conclude that S-gears reduce noise emission more successfully than E-gears under different types of loading and at the same temperature level. Future studies in the operating behaviour of S-polymer gears could also cover noise measurement and different failures when compared to E-gears. Future experiments could include new methods of sound signal analysis at different gear temperatures.

## Figures and Tables

**Table 1:** Torque at Frequency Inverter 2 (FI 2).

Torque *T* [Nm]	FI 2 [Hz]	Speed *n* [rpm]
1.1	35.75	1599
1.3	35.32	1579
1.5	34.35	1547

**Table 2:** Main geometrical data of tested gears.

Magnitude	S-Gears	E-Gears
Module	*m_n_* = 1 mm	*m_n_* = 1 mm
Number of teeth	*z*_1_ = *z*_2_ = 20	*z*_1_ = *z*_2_ = 20
Flank angle of tool	α_n_ = 18°	α_n_ = 18°
Tooth width	*b*_1_ = *b*_2_ = 6 mm	*b*_1_ = *b*_2_ = 6 mm
Profile shift coefficient	*x*_1_ = *x*_2_ = 0	*x*_1_ = *x*_2_ = 0
Quality of teething	*Q* = 8	*Q* = 8
Height factor	*a_P_* = 1.66	–
Curvature exponent	*n* = 1.85	–

**Table 3:** Materials properties of tested polymers.

Parameter	Standard	Delrin 100 NC010	Zytel 103 HSL NC010
Name abbreviation		POM-H	PA66-HT
Elastic modulus	ISO 527	3100 MPa	3100 MPa
Tensile strength	ISO 527	69 MPa	85 MPa
Yield strain	ISO 527	25%	4.5%
Flexural modulus	ISO 178	2700 MPa	2800 MPa
CLTE	ISO 11359-1/-2	1.0·10^−4^/°C	1.1·10^−4^/°C
Melting temperature	ISO 11357-1/-3	178 °C	262 °C
Glass transition temperature	DIN53765	−70 °C	47 °C
Thermal conductivity	ISO 220776-4	0.3 W/(mK)	0.23 W/(mK)
Specific heat	ISO 22007-4	1.4 J/(g·K)	1.67 J/(g·K)
Density	ISO 1183	1.41 g/cm^3^	1.14 g/cm^3^

## Data Availability

The data presented in this study are available on request from the corresponding author.
